# Factors related to depression in older adults during the COVID-19 pandemic in two coastal regions of Peru: An analytical cross-sectional study

**DOI:** 10.12688/f1000research.70655.1

**Published:** 2021-09-22

**Authors:** Elena de Jesús Quispe Sáenz, José Fernando Salvador-Carrillo, Oriana Rivera-Lozada, CESAR ANTONIO BONILLA ASALDE

**Affiliations:** 1 ESCUELA DE MEDICINA HUMANA, UNIVERSIDAD PRIVADA SAN JUAN BAUTISTA, CHORRILLOS, LIMA, Peru; 2 Escuela de Medicina, Universidad Privada San Juan Bautista Chincha, Chincha, ICA, Peru; 3 South American Center for Education and Research in Public Health, UNIVERSIDAD PRIVADA NORBERT WIENER, LIMA, Peru

**Keywords:** COVID-19; mental health; depression; associated factors; older adult; Peru

## Abstract

**Background:** Mental health problems such as anxiety, depression and their aggravation have been studied extensively in the general population. However, there are few studies on depression in older adults and the few existing results may be contradictory, especially in the context of the COVID-19 pandemic. The aim of this study is to determine the factors associated with depression in older adults in two coastal regions of Peru during the COVID-19 pandemic.

**Methods:** This study uses an analytical cross-sectional design in a population of older adults, who participated in a non-governmental ambulatory social support program in Callao and Ica, two coastal regions of Peru. We administered an on-site structured questionnaire to record sociodemographic data, the Geriatric Depression Scale by Yesavage to measure depression, and the Barthel Index to assess physical function. In order to determine cognitive impairment as an exclusion criterion, the MEC-30 was used. The association between variables was assessed through contingency tables, using the odds ratio (OR) with its corresponding confidence interval (95% CI) and the X2 test. Finally, a binary logistic regression analysis was performed.

**Results:** Out of the 244 older adults surveyed, 39% had depressive symptoms, of which 28.3% (n=69) and 10.7% (n=26) were moderately and severely depressive, respectively. The findings significantly associated with the presence of depressive symptoms were being 76 years old or older [p=0.005, OR: 2.33, 95% CI: 1.29-4.20], not participating in weekly recreational activities [p=0.004, OR: 2.28, 95% CI: 1.31-3.99] and the presence of comorbidities [p=0.026, OR: 1.88, 95% CI: 1.07-3.29].

**Conclusion:** There are few studies exploring depression in older adults during the COVID-19 pandemic; this research shows the importance of mental health care in this population and, particularly, of those who are 76 or older because they suffer from comorbid conditions and have interrupted recreational activities.

## Introduction

Prior to the COVID-19 pandemic, the world was concerned with the aging population, a situation that was by no means alien to developing countries. ^1^^,^^2^ In the same context, the relationship between older age and health status is well known; the former is the expression of a process that occurs throughout life and involves changes in biopsychosocial and cognitive aspects; the latter is a biopsychosocial construct based on a variety of circumstances that have to do with physical and mental adaptation and the environment. ^3^^,^^4^

Depression is a common condition in older adults. According to the World Health Organization, it is the second cause of global morbidity, after cardiovascular diseases, and has a multifactorial origin. ^5^ Depression has a diverse clinical manifestation that is difficult to identify, which causes a decrease in the quality of life and an increase in suffering, often leading to fatal outcomes. ^6^^**–**^^8^

A study conducted in Peru determined depressive disorders in older adults in rural communities of high Andean regions during the period 2013-2017. This identified 40.2% of people suffering some kind of depression according to the Yesavage questionnaire. ^9^ However, a subsequent study showed an estimated prevalence of depression in older adults of 14% and identified risk factors such as female gender, being 75 or older, living without a partner, low income, rurality and having at least one disability. ^10^

Peru has a population made up of 29,381,884 inhabitants, where older adults represent 6.2%. Callao, the country's first seaport, is one of the smallest and most densely populated regions, as well as one of the most industrialized after Lima, the capital city. On the other hand, Ica is one of the most productive regions, with less total poverty and extreme poverty than the national average. Both regions are home to 6.3% of the country's elderly. ^11^

The COVID-19 pandemic, which started in China at the end of December 2019, also affected Peru. By December 20, 2020, there were 998,475 confirmed cases and 37,173 deaths; by January 10, 2021, there were already 1,037,350 cases and 38,335 deaths, placing Peru in the 17th place out of 20 countries that reported the highest number of cases in the world. ^12^

Mental health can be affected as a consequence of events related to natural disasters, armed conflicts, threats to individual or collective health, among others. Older adults are a high-risk group and highly vulnerable to these situations. ^13^ As a consequence, the quality of life of older adults in a pandemic scenario is a cause for concern, characterized by high levels of uncertainty which, together with voluntary or mandatory confinement, physical and social isolation, can cause emotional imbalance. However, these are not the only factors that can influence this situation: comorbidities, degree of dependency, exposure to family abuse, loneliness, low educational level, lack of income or insufficient income can contribute to the deterioration of mental health, causing pathologies that are a challenge for public health. ^14^^**–**^^18^

The purpose of this study was to determine the factors associated with depression in older adults, under COVID-19 pandemic conditions, producing evidence to guide interventions that contribute to the improvement of the care and quality of life of this vulnerable population.

## Methods

### Study design and population

This is an analytical cross-sectional study. The population consisted of 263 older adults who received ambulatory care through the Community Promotion Development Liberation (COPRODELI) social program. This is a nonprofit association of Spanish origin that helps high-risk populations in marginal urban areas of two regions of Peru, Ica and Callao.

A non-probabilistic convenience sample of older adults with the following inclusion criteria was included: (i) aged 60 years or older, (ii) enrolled in the COPRODELI older adult social program, (iii) with no previous psychiatric diagnosis or with neurological diseases that would prevent them from participating in this study, (iv) receiving any type of psychiatric/neurological treatment, (v) not presenting with cognitive impairment (score greater than 23 points) according to the Mini-Mental Status Examination ( *Mini-Examen Cognoscitivo*, MEC-30) ^19^ and (vi) voluntarily signing the informed consent.

### Data collection

Data were collected during December 20, 2020 to January 10, 2021. This period was included in the second wave of the COVID-19 pandemic in Peru.

The instruments used were not modified and the instructions of the authors who designed the instrument were followed. The printed questionnaires were applied by the research team on site at the facilities of the COPRODELI association.

Biosecurity measures to avoid COVID-19 transmission were respected at all times during data collection. The research assistants were trained on the correct administration of the instruments and related ethical issues. Data collection was supervised by the authorities of the COPRODELI association and by the head researcher *. A copy of the questionnaire can be found in the Extended Data.*

### Variables and instruments

Data were collected using a structured questionnaire to record sociodemographic data such as gender, age, educational level, area of residence, family/social support, lifestyles and impact of the COVID-19 pandemic. Thus, we used the Geriatric Depression Scale, created by Yesavage20, to assess depressive symptoms; the Barthel Index17 to assess physical function; and the MEC-3021 to evaluate cognitive impairment.

The Yesavage Geriatric Scale and the Barthel Index showed a reliability coefficient of 0.653 and 0.630, respectively, measured through the KR-20 test.

### Statistical analysis

Two data entries were entered in Excel in parallel and independently and were analyzed in IBM SPSS version 22, using the Universidad Privada San Juan Bautista’s license, Lima, Peru. All questionnaires were coded, and data processing was supervised by the principal investigator **.** Data analysis was performed in four phases. The first phase included the descriptive analysis of the variables, using frequencies for the categorical variables. Analysis of the total population and by region were considered for subsequent analyses. In the second phase, a quantitative analysis of the scores obtained by the participants was performed. The scores showed a non-parametric distribution (Kolmogorov- Smirnov test: p < 0.001 in all cases) with the median value and the interquartile range (IQR). The Mann-Whitney U test was used to compare the scores between the populations of the regions of Ica and Callao. The third phase considered a bivariate analysis, where the association between variables was evaluated by means of contingency tables, using the odds ratio (OR) with its corresponding confidence interval (95% CI) and the X2 test. Finally, in the third phase, a binary logistic regression analysis was performed to determine the factors associated with depressive symptoms in the older adult population. Statistically significant was considered when the p value lower than 0.05.

### Ethical considerations

Ethical standards were respected throughout the research process. The Institutional Research Ethics Committee of San Juan Bautista University approved the study protocol and informed consent procedures with file Number 185-2020-CIEI-UPSJB. The questionnaires were administered on site and written informed consents were obtained. We considered that participants should be older adults with orientation to time, space and person, without either cognitive impairment, a limiting neurological or psychiatric diagnosis/treatment who do not have hearing impairment. In addition, biosafety prevention and control protocols were strictly followed to avoid the spread and infection of COVID-19 by respondents and survey personnel (use of double masks, face shields and a distance of 1.5 meters), in accordance with national legal standards and international technical documentation in force.

Before completing the questionnaires, participants were informed about the purpose of the study and the voluntary nature of their participation. The surveys were anonymous, and the data were treated with strict confidentiality. Older adults who presented with depressive symptoms were reported to the COPRODELI association so that they could be considered in the management of their social care.

## Results

### Characteristics of the participants

**Figure 1.**
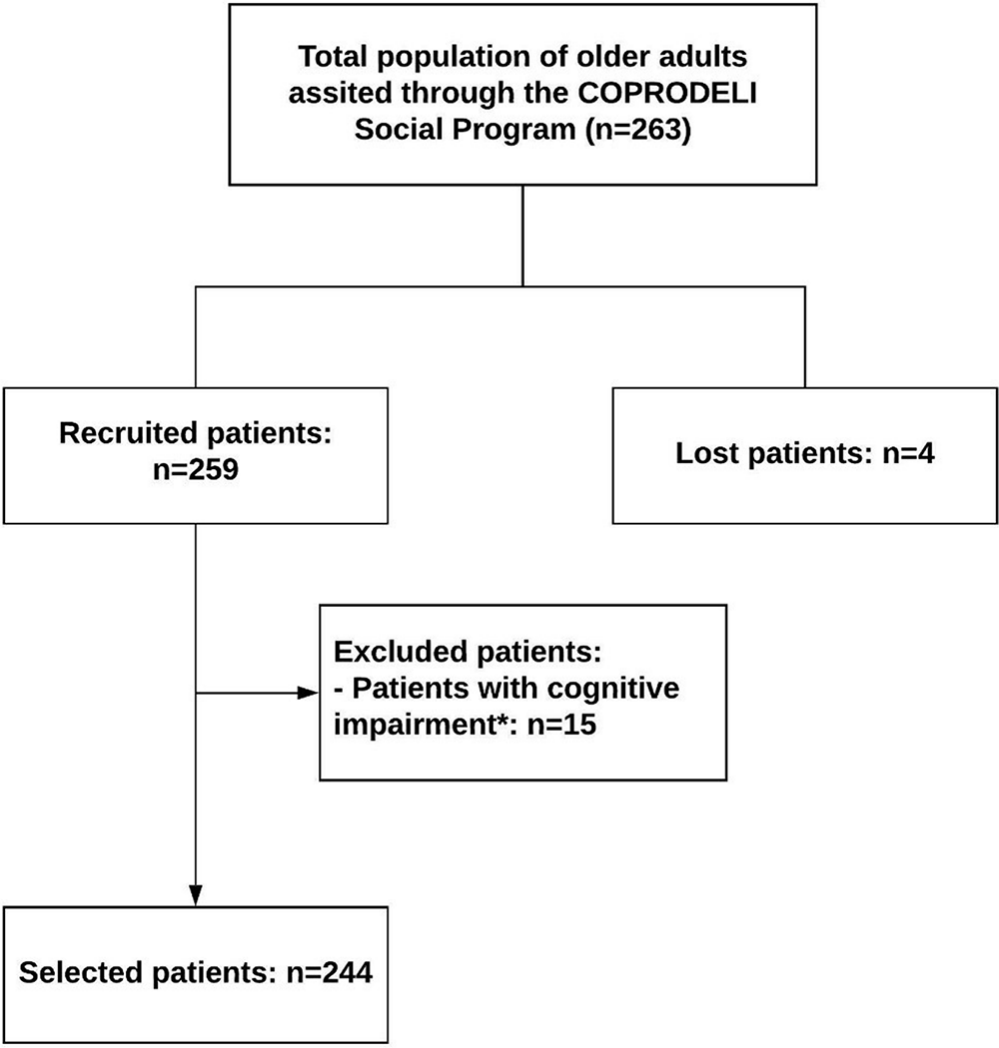
Recruitment and selection process of older adult participants enrolled in the COPRODELI Social Program during the COVID-19 pandemic, Peru. *Patient with cognitive impairment: score ≤ 23 on the Mini-Mental Status Examination -30.

A total of 259 older adults enrolled in the COPRODELI Social Program were recruited. According to the selection criteria, a total of 15 participants were excluded: older adults with cognitive impairment (score ≤ 23, MEC-30). A total of 244 participants were considered ( Figure 1).

The mean age of the older adults was 73 (± 6.0) years old (range: 60-95). Of the total, 61.9% (n = 151) identified themselves as female, 36.5% (n = 89) were married, 77.9% (n = 190) completed school education, 53.1% (n = 130) were from urban areas and 60.7% (n = 148) resided in Callao. On the other hand, 28.3% (n = 69) and 10.7% (n = 26) of the participants presented with moderate and severe depressive symptoms, respectively. The other characteristics were described in Table 1.

**Table 1.** Characteristics of older adults in the COPRODELI Social Program during the COVID-19 Pandemic, Peru.

Characteristics	n	%
**Sociodemographic factors**		
Gender		
Man	93	38.1
Woman	151	61.9
Age		
60-75	176	72.1
76 or older	68	27.9
Civil status		
Married	89	36.5
Widow	54	22.1
Single	88	36.1
Divorced	13	5.3
Residence area		
Urban	130	53.1
Rural	114	46.9
Level of education		
Incomplete schooling	54	22.1
Complete schooling	190	77.9
Region		
Ica	96	39.3
Callao	148	60.7
**Family/social support**		
Who do you live with?		
With family	147	60.2
Alone	97	39.8
Social programs support		
Only COPRODELI	189	77.5
More than one social program	55	22.5
**Lifestyle**		
Weekly recreational activities		
No	108	44.3
Yes	136	55.7
Presence of comorbidity		
No	100	41.0
Yes	144	59.0
Hypertension	50	20.5
Diabetes	18	7.4
Arthrosis	14	5.7
Others	109	44.7
**Impact of the COVID-19 pandemic**		
Death of a close relative due to COVID-19		
No	186	76.2
Yes	58	23.8
COVID-19 test		
Negative/Without a diagnosis	221	90.6
Positive	23	9.4
**Physical function according to the Barthel index**		
Independency	169	69.3
Low dependency	35	14.3
Moderate dependency	36	14.8
Severe dependency	4	1.6
**Depressive symptoms according to the Yesavage Scale**		
Normal	149	61.1
Moderate	69	28.3
Severe	26	10.6

### Scores for depressive symptoms

**Figure 2.**
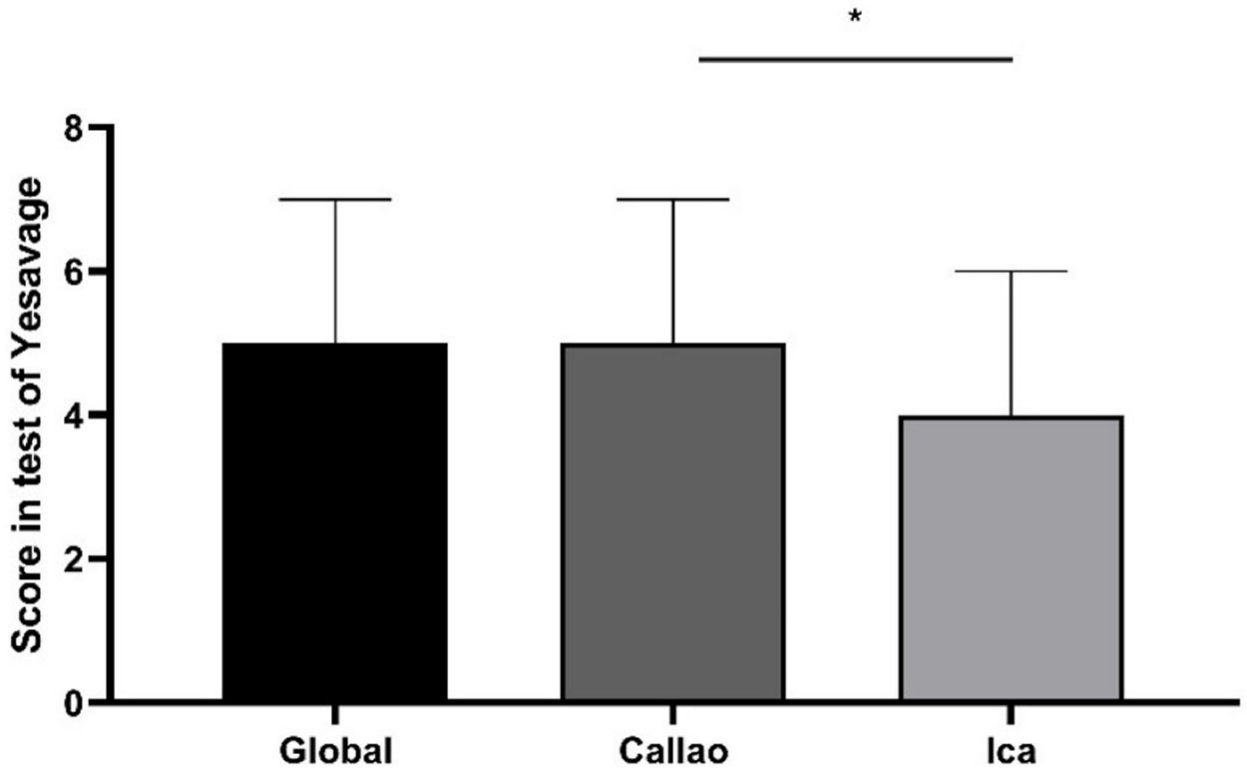
Scores obtained by older adult participants on the Yesavage Scale during the COVID-19 Pandemic in two regions of Peru. Error bars represent the median and interquartile range, respectively. *p = 0.028 in the Mann-Whitney test.

The score obtained from the total number of participants had a median of 5 [IQR: 3-7] on the Yesavage Scale. We found 0 as a minimum value and the maximum value was 14 points. In addition, in Ica, the participants obtained a median score of 4 [IQR: 2-6] and in Callao, 5 [IQR: 3-7]. A statistical difference (p = 0.028) was found between the obtained scores among participants from the region of Ica compared to those from Callao ( Figure 2).

### Bivariate analysis

An exploratory analysis between all the co-variables and the presence of depressive symptoms in all the older adult participants and by region (Callao and Ica) was performed. It was demonstrated that there was a significative association between the population older than 75 and the presence of depressive symptoms with respect to the total population (p = 0.005, OR: 2.23, 95% CI: 1.26-3.94). Similarly, being older than 75 was associated significantly with being part of the population of the region of Callao (p = 0.01, OR: 2.61, 95% CI: 1.24-5.48). When the two regions were analyzed in the total population, it was found that belonging to Callao was associated with the presence of depressive symptoms (p = 0.047, OR: 1.73; 95% CI: 1.01-2.95). Interestingly, it was also found that participating in more than one social program was a protective factor solely for the inhabitants of the Callao region (p = 0.005; OR: 0.24, 95% CI: 0.08-0.69).

On the other hand, not engaging in weekly recreational activities and presenting with comorbidities were also factors associated with depressive symptoms for the total population (p = 0.003, OR: 2.20, 95% CI: 1. 29-3.77 and p = 0.017, OR: 1.92, 95% CI: 1.12-3.29, respectively) and for the inhabitants of the region of Ica (p = 0.006; OR:3.45; 95% CI: 1.40-8.51 and p = 0.006, OR: 3.45, 95% CI:1.40-8.51, respectively).

Finally, physical function according to the Barthel index was also statistically associated with depression in older adults for the total population and for the region of Callao (p = 0.026 and p = 0.015, respectively), but only moderate-severe dependency had a significant risk in both populations (total: OR: 2.59, 95% CI: 1.28-5.23 and Callao: OR: 2.81; 95% CI: 2.81-6.54). In the low dependency category, no significant risk for developing depressive symptoms was found for any group (p > 0.05) ( Table 2).

**Table 2.** Bivariate analysis of depression in older adults enrolled in the COPRODELI Social Program during the COVID-19 pandemic, Peru.

Characteristic	Presence of depressive symptoms * (n = 95)	Absence of depressive symptoms * (149)	Total (n = 244)		Ica (n = 96)		Callao (n = 148)	
	n (%)	n (%)	p-value	OR [95% IC]	p-value	OR [95% IC]	p-valor	OR [95% IC]
**Sociodemographic factors**								
Gender								
Man	37 (39.8)	56 (60.2)	0.831	0.94 [0.55-1.60]	0.597	0.791 [0.33-1.88]	0.955	0.98 [0.49-1.93]
Woman	58 (38.4)	93 (61.6)						
Age								
60-75	59 (33.5)	114 (66.5)	**0.005**	**2.23 [1.26-3.94]**	0.209	1.80 [0.71-4.59]	**0.01**	**2.61 [1.24-5.48]**
76	36 (52.9)	32 (47.1)						
Civil status								
Married	36 (40.4)	53 (59.6)	0.71	0.90 [0.53-1.54]	0.698	0.84 [0.35-2.01]	0.629	0.84 [0.42-1.68]
Single/widow/divorced	59 (38.1)	96 (61.9)						
Residence area								
Urban	48 (36.9)	82 (63,1)	0.49	1.19 (0.71-2.00)	0.34	1.53 [0.63-3.71]	0.58	1.26 [0.62-2.33]
Rural	47 (41.2)	67 (58.8)						
Level of education								
Incomplete schooling	15 (27.8)	39 (72.2)	0.06	1.86 [0.96-3.60]	0.105	2.88 [0.77-10,77]	0.189	1.69 [0.76-3.72]
Complete schooling	80 (42.1)	110 (57.9)						
Region								
Ica	30 (31.3)	66 (68.8)	**0.047**	**1.73 [1.01-2.95]**	NA	NA	NA	NA
Callao	65 (43.9)	83 (56.1)						
**Family/social support**								
Who do you live with?								
With my family	61 (41.5)	86 (58.5)	0.312	0.761 [0.44-1.29]	0.153	0.51 [0.20-1.28]	0.873	0.94 [0.48-1.84]
Others	34 (35.1)	6 (64.9)						
Social programs support								
Only COPRODELI	79 (41.8)	110 (58.2)	0.08	0.57 [0.29-1.09]	0.353	1.54 [0.61-3.87]	**0.005**	**0.24 [0.08-0.69]**
More than one social program	16 (29.1)	39 (70.9)						
**Lifestyle**								
Weekly recreational activities								
Yes	31 (28.7)	77 (71.3)	**0.003**	**2.20 [1.29-3.77]**	**0.028**	**2.71 [1.01-6.69]**	0.071	1.85 [0.94-3.64]
No	64 (47.1)	72 (52.9)						
Presence of comorbidity								
No	30 (30.0)	70 (70.0)	**0.017**	**1.92 [1.12-3.29]**	**0.006**	**3.45 [1.40-8.51]**	0.783	1.10 [0.54-2.24]
Yes	65 (45.1)	79 (54.9)						
**Impact of the COVID-19 pandemic**								
Death of a family member due to COVID-19								
No	73 (60.8)	113 (39.2)	0.85	0.94 [0.51-1.73]	0.892	0.92 [0.32-2.71]	0.794	0.90 [0.43-1.98]
Yes	22 (37.9)	36 (62.1)						
COVID-19 test								
Negative/without a diagnosis	84 (38.0)	137 (62.0)	0.35	1.49 [0.63-3.54]	0.15	3.55 [0.56-22.49]	0.962	1.02 [0.38-2.76]
Positive	11 (47.8)	12 (52.2)						
**Physical function according to the Barthel index**								
Independency	58 (34.3)	111 (65.7)	**0.026**	Ref.	0.729	Ref	**0.015**	Ref
Low dependency	14 (40.0)	21 (60.0)		1.26 [0.60-2.69]		1.349 [0.40-4.50]		1.21 [0.46-3.16]
Moderate-severe dependency	23 (57.5)	17 (42.5)		**2.59 [1.28-5.23]**		1.61 [0.41-6.33]		**2.81 [1.19-6.54]**

Percentage values are shown according to the values of the variables horizontally. NA: Not applicable; CI: Confidence interval; Ref: Reference. *Distributions calculated over the total number of participants.

### Logistic regression analysis

For multivariate analysis, the model was constructed with the variables that were significantly associated with the presence of depressive symptoms in the bivariate analysis for the total population. Being older than 75 (p = 0.005, OR: 2.33, 95% CI: 1.29-4.20), not participating in weekly recreational activities (p = 0.004, OR: 2.28, 95% CI: 1.31-3.99) and the presence of comorbidities (p = 0.026, OR: 1.88, 95% CI: 1.07-3.29) were factors associated with the presence of depressive symptoms in our studied sample ( Table 3). On the other hand, physical function according to the Barthel index and belonging to any region did not have significant association with the presence of depressive symptoms (p > 0.05).

**Table 3.** Logistic regression analysis of depression in older adults enrolled in the Social Program-COPRODELI during the COVID-19 Pandemic, Peru.

Characteristic	Total population	
	p-value	Adj ^a^ OR (95% IC)
Age		
60-75	0.005	Ref.
76 or older		2.33 [1.29-4.20]
Region		
Ica	NS	Ref.
Callao		NS
Social program support		
Only COPRODELI	-	-
More than one social program	-	-
Weekly recreational activities		
Yes	0.004	Ref.
No		2.28 [1.31-3.99]
Presence of comorbidity		
No	0.026	Ref.
Yes		1.88 [1.07-3.29]
Physical function according to the Barthel index		
Independency		Ref.
Low dependency	NS	NS
Moderate-severe independency	NS	NS

^a^ Adjusted by age, region, weekly recreational activities, presence of comorbid conditions and physical function according to the Barthel index.

## Discussion

During the second wave of the COVID-19 pandemic, ten months after the identification of the first case in Peru on March 05, 2020, depression was investigated in a sample of older adults participating in a non-governmental ambulatory support program. The findings significantly associated to the presence of depressive symptoms were being 76 or older, not participating in weekly recreational activities and the presence of comorbid conditions.

Regarding the COVID-19 pandemic, there are innumerable studies in the general population on mental health problems such as anxiety, depression and its aggravation, ^22^ but there are few studies focused on depression in older adults and the few existing results may be contradictory. ^23^ In addition, this research was one of the few studies where the instrument was administered on site.

The participants from the region of Callao showed higher levels of depression than those from the region of Ica. Probably, this can be explained due to the difference in the economic income of both populations. There exist studies that showed that socioeconomic level and residence area are protective factors for health, especially when associated with educational level and family income. ^24^^,^^25^ In Peru, Saenz *et al.* ^9^ and Martina *et al.* ^10^ found similar results about the association between depression in older adults and economic income in periods prior to the pandemic, which would permit us to understand the differences we found in our study. It is important to mention the lack of research regarding economic income and depression in older adults in the context of COVID-19 pandemic, which implies a challenge for future research.

The pandemic represents a serious public health issue, especially in older adults due to the association between comorbidities and high mortality, ^26^ in addition to the increased risk of depression ^27^ that affects them disproportionately due to the scarce social contact outside the home and with family or close friends. Some of them depend on the support of voluntary services or social assistance, but it can affect even more those who are already alone, isolated or reclusive. ^28^^,^^29^ An additional aspect related to the COVID-19 pandemic has to do with the health care approach implemented in terms of confinement, physical and social distancing, together with the loss of family and friends, including the alarming news broadcast by the media that increase the risk of mental health problems, especially in this population group. ^13^

The analysis by age group in this study shows significant differences in the group aged 76 and older, similar to what was found in the pre-pandemic period in Peru. ^9^ These findings are important to highlight because depressive disorders are more frequent in young adults, ^5^ a situation that continues in the context of the COVID-19 pandemic. ^30^ Age is an important sociodemographic variable in mental health, with the group of young adults showing higher levels of anxiety and depression compared to older adults. This is because they are less likely to have lived stressful experiences and to be unaccustomed to lower levels of social interaction, hence their low resilience. ^31^ In contrast, a study in China and India found that mental health problems are more frequent among older adults, especially the elderly, because they are more fragile, vulnerable and affected by multimorbidity. ^32^ It is within this last context that the explanation of what may be happening with the study population in this research is inserted.

Stanton ^33^ stated that during the pandemic, as a consequence of physical and social distancing, older adults dramatically decreased their recreational and daily activities, which increased their psychological distress. This situation makes it necessary to implement strategies to promote physical activity. In this regard, the findings of this research confirm the above.

Linked to the above, the findings are consistent with other studies that report more signs of depression in people with comorbidities. ^17, 29, 34, 35^

It is important to highlight that the descriptive data collected in this study, in comparison with those conducted during the pandemic in other countries, indicate that 38% of older adults reported moderate to severe depression, which is higher than the 16.8% reported in the general population in China ^36^ and 19.1% that reported prevalence of moderate to severe depression in Australia. ^33^ Another study conducted in China in the general population showed that 14.6% had depression, which was lower than what was reported in studies of other health emergencies. These differences can be explained due to the rapid and energetic government response to guarantee safe care. Other explanations are based on the level of knowledge of COVID-19 of citizens due to the rapid dissemination of information that reduced public panic and the implementation of a system of psychological assistance. ^37^ Something similar was found in an investigation in Spain, which identified less emotional distress in the elderly with no differences between men and women. ^23^

Although in the USA a nationally representative study showed that depression had more than a threefold increase in the general population and double increase in the population aged 60 years and older during the COVID-19 pandemic. However, these results were less than what is reported in this study. ^14^

In comparison with research on depression in older adults in Peru, in the pre-pandemic period, the study by Martina *et al.*, ^10^ closer to this study, showed that depression was lower by 24 percentage points, unlike the study by Saenz *et al*., ^9^ conducted exclusively with a rural high Andean population, where the results are similar to those presented here, although it is noteworthy that slightly more than 50% of the participants in our study came from urban areas. Both studies showed rural origin as a risk factor for depression in older adults. The results of this research allow us to hypothesize that rurality alone does not explain these differences, since they could be justified by the economic conditions of poverty and extreme poverty in which these populations live and which may eventually be aggravated by traumatic conditions, such as what happened during the COVID-19 pandemic.

Contrary to the results of research indicating that loneliness is a risk factor for depression, ^23^^,^^38^ in this study, 60.2% of the older adults lived with their families and 62.3% maintained frequent telephone communication with their families. The explanation for this difference in the high presence of depression is the known fact that living together and greater socialization during the pandemic increases the risk of contagion and therefore, older adults in these cases have greater psychological pressure: feeling afraid of contracting the disease and dying, as well as the possibility that other close relatives may be affected by COVID-19. ^30^

Although the instrument was applied ten months after the start of the pandemic, during the second wave, when uncertainty is expected to be lower due to the time elapsed, the levels of depression were higher than expected compared to other studies, which may be explained by the excess of sensationalist news and rumors or misinformation about the pandemic. ^39^

Research in the general population has found that anxiety disorders and depression were more frequent in women in all age groups. During the COVID-19 pandemic, these similarities continued, and they are even being identified as the strongest predictor of post-traumatic stress symptoms after pandemics. ^29^^,^^32^^,^^40^ This is in agreement with our findings, where 61.9% of the studied sample were women. This situation allows us to propose as a new hypothesis: given that women are more common in all age groups and that the feeling of loss of a family member is more accentuated among them, these may lead to higher levels of depression.

The results of this study should be analyzed in light of some limitations; certain results are based on descriptive analysis; therefore, causality cannot be inferred. Moreover, the study sample was obtained from a population receiving ambulatory social support and may not be representative of the general population; additionally, the study could be exposed to sampling bias.

This research could serve as a basis for future studies to analyze the behavior of older adults under pandemic conditions and to determine how the measures implemented to ensure their well-being, in the face of COVID-19, cannot lead to mental health problems such as depression.

This research is one of the few studies exploring depression in older adults. The results allowed us to infer that mental health care is fundamental in this population and, specially, in those who are 76 and older because they suffer from comorbidities and have interrupted recreational activities. There is a need for protection of these highly vulnerable people at high risk of developing depression. There should be health promotion interventions and prevention of mental illness, with emphasis on the first level of care that allows the attenuation of the effects of compulsory confinement, physical and social distancing, in addition to the excess of sensationalist information.

## Data availability

### Underlying data

Zenodo: Factors related to depression in older adults during the COVID 19 pandemic in two coastal regions of Peru: An analytical cross-sectional study. https://doi.org/10.5281/zenodo.5498990. ^41^

This project contains the following underlying data:

- DATABASE_V4. 09-09.xlsx (dataset)

### Extended data

Zenodo: Factors related to depression in older adults during the COVID 19 pandemic in two coastal regions of Peru: An analytical cross-sectional study. https://doi.org/10.5281/zenodo.5498990. ^41^

This project contains the following extended data:

- FICHA DE RECOLECCIÓN DE DATOS 9-09.docx (a copy of the questionnaire)

Data are available under the terms of the Creative Commons Attribution 4.0 International license (CC-BY 4.0).
