# Supplementary material for: Factors related to depression in older adults during the COVID-19 pandemic in two coastal regions of Peru: An analytical cross-sectional study
Source: F1000Res. 2022 Mar 10;10:958. Originally published 2021 Sep 22. [Version 3] doi: 10.12688/f1000research.70655.3 (PMC8924554; doi:10.12688/f1000research.70655.3)
Supplement: Peer review history

# Peer review: Reviewer response for version 3

**DOI:** 10.5256/f1000research.122069.r127030

**Published:** 2022-03-15

**Authors:** Julio Torales

**Affiliations:** 1 Department of Psychiatry, School of Medical Sciences, National University of Asunción, San Lorenzo, Paraguay

The authors have incorporated the recommendations from the review process. I believe that the article is now clearer and more useful to readers. Thank you very much.

Is the work clearly and accurately presented and does it cite the current literature?

Yes

If applicable, is the statistical analysis and its interpretation appropriate?

Yes

Are all the source data underlying the results available to ensure full reproducibility?

Yes

Is the study design appropriate and is the work technically sound?

Yes

Are the conclusions drawn adequately supported by the results?

Yes

Are sufficient details of methods and analysis provided to allow replication by others?

Yes

Reviewer Expertise:

Psychiatry, Mental Health, Special populations,

I confirm that I have read this submission and believe that I have an appropriate level of expertise to confirm that it is of an acceptable scientific standard.

# Peer review: Reviewer response for version 2

**DOI:** 10.5256/f1000research.121057.r123122

**Published:** 2022-02-21

**Authors:** Julio Torales

**Affiliations:** 1 Department of Psychiatry, School of Medical Sciences, National University of Asunción, San Lorenzo, Paraguay

This is a very interesting article, appropriate to the current times. The authors have presented a clear and concise second revised version. Nevertheless, I would like to recommend to the authors:

1. To specify whether or not the participants were diagnosed with COVID-19. This is important since COVID-19 infection can cause direct effects on the brain and indirect effects through cytokine storm, which can generate long-term negative mental health outcomes. This may be a confounding factor to consider.
2. To elaborate further on what kind of interventions should be put in place, by governments and health authorities, to promote and protect the mental health of this vulnerable population during and after the pandemic.

Is the work clearly and accurately presented and does it cite the current literature?

Yes

If applicable, is the statistical analysis and its interpretation appropriate?

Yes

Are all the source data underlying the results available to ensure full reproducibility?

Yes

Is the study design appropriate and is the work technically sound?

Yes

Are the conclusions drawn adequately supported by the results?

Yes

Are sufficient details of methods and analysis provided to allow replication by others?

Yes

Reviewer Expertise:

Psychiatry, Mental Health, Special populations,

I confirm that I have read this submission and believe that I have an appropriate level of expertise to confirm that it is of an acceptable scientific standard, however I have significant reservations, as outlined above.

# Response

**Published:** 2022-03-01

**Authors:** CESAR ANTONIO BONILLA ASALDE

**Affiliations:** SAN JUAN BAUTISTA UNIVERSISTY, Peru

**Reply to reviewer**

- To specify whether or not the participants were diagnosed with COVID-19. This is important since COVID-19 infection can cause direct effects on the brain and indirect effects through cytokine storm, which can generate long-term negative mental health outcomes. This may be a confounding factor to consider.

**This was added to the discussion**

 Through this study, we found that 9.4% (n=23) participants reported having a positive COVID-19 diagnosis. In Pearson's Chi-squared test analysis, no statistical association was found between the development of depression and COVID-19 diagnosis (p>0.05). It is possible that some patients may have had COVID-19, been asymptomatic, and not had the appropriate diagnosis for COVID-19; however, in the proposed design for this study, it would not have been possible to identify these participants.

  

- To elaborate further on what kind of interventions should be put in place, by governments and health authorities, to promote and protect the mental health of this vulnerable population during and after the pandemic.

**This was added to the discussion**

 Definitely, depression in older adults is a challenge for public health and health systems; hence, there is a need for governments and health authorities to improve care in this serious risk and high vulnerability population during and after traumatic situations such as the COVID 19 pandemic.  Consequently, prevention and promotion strategies are proposed, based on the evidence generated by this study. We can emphasize those interventions that promote the implementation of physical and recreational activity to avoid the development of chronic conditions such as depression and reduce their impact on health and quality of life. It is also advisable to include interventions of digital health and self-care literacy that favor cognitive and physical stimulation in the performance of daily life actions and in the relationship with family and friends. As depression is a multifactorial condition, the approach should be comprehensive, based on continuous care, focused and adapted to the needs of older adults, contributing to active aging.  Another aspect to be explored is the preparation of health personnel in terms of old age, aging and their approach strategies due to the stigmatization in the care of older adults. This preparation will allow real comprehensive health care, which contributes toward the improvement of competencies in working with older adults and increases the social value of the intervention. It should not be forgotten to close gaps in knowledge through research, generating national evidence to improve the protective factors for older adults’ mental health.

# Peer review: Reviewer response for version 2

**DOI:** 10.5256/f1000research.121057.r123028

**Published:** 2022-02-10

**Authors:** Eric Kam-Pui Lee

**Affiliations:** 1 School of Public Health and Primary Care, Faculty of Medicine, The Chinese University of Hong Kong, Hong Kong, China

I have no further comments.

Is the work clearly and accurately presented and does it cite the current literature?

Yes

If applicable, is the statistical analysis and its interpretation appropriate?

Yes

Are all the source data underlying the results available to ensure full reproducibility?

Yes

Is the study design appropriate and is the work technically sound?

Yes

Are the conclusions drawn adequately supported by the results?

Yes

Are sufficient details of methods and analysis provided to allow replication by others?

Yes

Reviewer Expertise:

mindfulness, hypertension, primary care, public health

I confirm that I have read this submission and believe that I have an appropriate level of expertise to confirm that it is of an acceptable scientific standard.

# Peer review: Reviewer response for version 1

**DOI:** 10.5256/f1000research.74257.r97843

**Published:** 2021-10-27

**Authors:** Eric Kam-Pui Lee

**Affiliations:** 1 School of Public Health and Primary Care, Faculty of Medicine, The Chinese University of Hong Kong, Hong Kong, China

Background:

1. Consider stating a pre-defined hypothesis in the introduction.

Methods:

1. How is the sample size arrived at?
2. How did you measure family/social support? I think there are standardized questionnaires for these parameters. Same with lifestyle.
3. How did you deal with missing data? Any sensitivity analysis?

Discussion:

1. Can discuss the external validity of the study. Do these patients who are recruited from a social program represent the general population?
2. There are unmeasured important variables (this is almost inevitable for cross-sectional studies) and this can be discussed. E.g. the sleep quality, the exercise level - all of these can determine the presence of depression.
3. All the findings can be bidirectional e.g. people with more physical diseases are more prone to depression, but people with depression can have more physical problems. The same goes with recreational activities.
4. So what's the implication? Should we focus on screening people who are aged over 75 for depression? By who? Routinely?

Minor:

- This manuscript will benefit from English editing.

Is the work clearly and accurately presented and does it cite the current literature?

Yes

If applicable, is the statistical analysis and its interpretation appropriate?

Yes

Are all the source data underlying the results available to ensure full reproducibility?

Yes

Is the study design appropriate and is the work technically sound?

Yes

Are the conclusions drawn adequately supported by the results?

Yes

Are sufficient details of methods and analysis provided to allow replication by others?

Yes

Reviewer Expertise:

mindfulness, hypertension, primary care, public health

I confirm that I have read this submission and believe that I have an appropriate level of expertise to confirm that it is of an acceptable scientific standard, however I have significant reservations, as outlined above.

# Response

**Published:** 2022-02-04

**Authors:** CESAR ANTONIO BONILLA ASALDE

**Affiliations:** SAN JUAN BAUTISTA UNIVERSISTY, Peru

Dear reviewer,

 Please find attached the response to your comments, which have been incorporated in the paper. We appreciate your comments.

**Background:**

- **Consider stating a pre-defined hypothesis in the introduction.**

It was considered in the introduction:

 The aim of this study was to determine the factors associated with depression in older adults, under COVID-19 pandemic conditions. It is hoped to characterise the factors that are associated with depression in this vulnerable population in order to generate scientific evidence that will allow the design of more specific interventions that contribute to the improvement of care and quality of life in older adults.

**Methods:**

1. **How is the sample size arrived at?**

For the present study, the entire population of older adults receiving outpatient support through the COPRODELI social programme was considered. Since the total universe of patients (n=263) is not very large, all patients were invited in order to have an adequate number of participants so as not to affect the statistical power in the logistic regression analyses.

  

- **How did you measure family/social support? I think there are standardized questionnaires** **for these parameters. Same with lifestyle.**

The parameters social/family support and lifestyle contain the variables "if you lived alone during the pandemic", "if you belonged to one or more social support programmes", and, "if you do weekly recreational activities" and "if you have any comorbidity", respectively. These variables were considered to assess social support and lifestyle because they are adapted to the social context experienced by older adults in Peru during the COVID-19 pandemic. Although there are validated instruments to assess these parameters, these were not the main study variables, so only these variables were considered in dichotomous form in order to have a concise questionnaire that can be applied to older adults.

  

- **How did you deal with missing data? Any sensitivity analysis?**

A sensitivity analysis was not performed because all recruited participants responded satisfactorily to all questions in the survey administered. This can be corroborated in table 1. Characteristics of older adults in the COPRODELI Social Programme during the COVID-19 pandemic in Peru, where it can be seen that all data were obtained from the 244 participants.

**Discussion:**

- **Can discuss the external validity of the study. Do these patients who are recruited from a** **social program represent the general population?**

**Methods:**

 (i) aged 60 years or older living in the community and not in asylums or other similar institutions (ii) enrolled in the COPRODELI older adult social program where they participate voluntarily

 Discusión:

 During the second wave of the COVID-19 pandemic, ten months after the identification of the first case in Peru on March 05, 2020, depression was investigated in a sample of older adults participating in a non-governmental ambulatory support program. The findings significantly associated to the presence of depressive symptoms were being 76 or older, not participating in weekly recreational activities and the presence of comorbid conditions. The results are generalizable to older population that comes from urban-marginal areas at high social risk, and we need to be precautious when extending the results to the general population in this age group.

  

- **There are unmeasured important variables (this is almost inevitable for cross-sectional studies) and this can be discussed. E.g. the sleep quality, the exercise level - all of these can** **determine the presence of depression.**

Discusión:

 Regarding the COVID-19 pandemic, there are innumerable studies in the general population on mental health problems such as anxiety, depression and its aggravation, ^22^ but there are few studies focused on depression in older adults and the few existing results may be contradictory. ^23^ In addition, this research was one of the few studies where the instrument was administered on site. Moreover, depression in older adults is, because of its nature, the result of the complex multidirectional interaction of a series of biological, psychological and social variables, expressed through clinical-epidemiological, cultural, economic and geographic aspects.

 The results of this study should be analyzed in light of some limitations; certain results are based on descriptive analysis; therefore, causality cannot be inferred. Moreover, the study sample was obtained from a population receiving ambulatory social support and may not be representative of the general population; additionally, the study could be exposed to sampling bias. Our study has considered variables that explain the multifactor nature of depression in older adults. However, we did not include quality of life, quality of sleep and level of physical exercise that could lead to the onset of depressive symptoms, which are particularly relevant issues to explore in future research.

  

- **All the findings can be bidirectional e.g. people with more physical diseases are more prone to depression, but people with depression can have more physical problems. The same** **goes with recreational activities.**

Stanton ^33^ stated that during the pandemic, as a consequence of physical and social distancing, older adults dramatically decreased their recreational and daily activities, which increased their psychological distress. This situation makes it necessary to implement strategies to promote physical activity. In this regard, the findings of this research confirm the above. It should be emphasized that participation in recreational activities or physical activity has a protective effect on depression. This favors better cognitive productivity while, at the same time, allows the alleviation of possible social isolation when interactive activities are carried out, increasing the perception of quality of life in older adults. In contrast, there is evidence that retirement significantly reduces the risk of suffering from health problems, which is not associated with a higher level of depression. The explanation is that it could be due to the decrease in work stress.

 Linked to the above, the findings are consistent with other studies that report more signs of depression in people with comorbidities. ^17,29,34,35  ^Arterial hypertension is the most important risk factor for developing cerebrovascular disease in the frontal lobe or putamen. However, it is not the only cause as there are other comorbidities such as heart disease, dyslipidemia, diabetes mellitus and insulin resistance. Older adults are at greater risk for developing these chronic diseases and all of them can contribute to the presence of depression 

 Depression is the third leading cause of disability in the world. Despite the burden of these disorders, their correlation with other medical conditions such as chronic diseases tends to be underestimated. Depression is a current public health problem in the world and a challenge for decision makers.  

 It should be added that we need to pay special attention to older adults with comorbidities. They may cause the decrease in their functional suitability to participate in recreational activities as these conditions may limit their physical capacity for exercise. 

  

- **So what's the implication? Should we focus on screening people who are aged over 75 for** **depression? By who? Routinely?**

The demographic and epidemiological transition in the world will lead to an increase in life expectancy at birth and an increase in chronic non-communicable diseases. These changes will accentuate the existence of a greater number of older adults in the coming years, and a high proportion of them, with depression. In this perspective, it is necessary to reorient public health strategies focused on older adults in order to respond to this problem. There is a clear need to strengthen mental health services and resources at the first level of care through an integral action.
